# Identification of *Shewanella putrefaciens* as a novel pathogen of the largemouth bass (*Micropterus salmoides*) and histopathological analysis of diseased fish

**DOI:** 10.3389/fcimb.2022.1042977

**Published:** 2022-10-17

**Authors:** Xinyu Jiang, Xiaoyu Wang, Lei Li, Chen Niu, Chao Pei, Lei Zhu, Xianghui Kong

**Affiliations:** Engineering Lab of Henan Province for Aquatic Animal Disease Control, College of Fisheries, Henan Normal University, Xinxiang, China

**Keywords:** *Shewanella putrefaciens*, *Micropterus salmoides*, virulence, water temperature, antibiotic resistance

## Abstract

The largemouth bass (*Micropterus salmoides*) is an economically important aquaculture species in China, and its production has increased rapidly in recent years. Although *Shewanella putrefaciens* is known to infect several fish species, its role in infecting *M. salmoides* is relatively unknown. Here, we isolated a gram-negative bacterial strain (termed XX2021) from farmed largemouth bass. Based on the results of 16S rRNA sequencing and phylogenetic analyses, the isolate was identified as *S. putrefaciens.* The virulence of XX2021 was dependent on water temperature, such as the LD_50_ values were 4.21×10^4^, 7.26×10^5^, and 2.47×10^6^ CFU/g fish weight at 10°C, 18°C, and 25°C, respectively. Four virulent genes—including *dksA, hem, lonR*, and *fur*—were screened through a PCR assay. The results of an antibiotic resistance test showed that XX2021 was sensitive to kanamycin, cefotaxime, doxycycline, sulfamethoxazole, florfenicol, tetracycline, and gentamicin; showed intermediate susceptibility to streptomycin, ampicillin, and norfloxacin; and was resistant to nalidixic acid and penicillin. XX2021-infected fish showed clinical symptoms typical of *S. putrefaciens* infection. In addition, we re-isolated XX2021 from infected fish and confirmed its identity using 16S rRNA sequencing. Histopathological changes were observed in the intestine, head kidney, spleen, and liver of diseased fish. This study presents the first report of the pathogenic effects of *S. putrefaciens* in farmed largemouth bass. Our findings may help develop effective disease control strategies for aquaculture fish and prevent disease outbreaks under low water temperatures.

## Introduction

*Shewanella putrefaciens* is a non-fermentative, motile, gram-negative bacillus with chief phenotypic attribute of H_2_S production (Holt et al., 2005). The species belongs to the genus *Shewanella*, named after the microbiologist James Shewan who had made outstanding contributions to the field of marine microorganisms (MacDonell and Colwell, 1985). In mammals, *Shewanella* spp. are associated with various infections—such as ear, skin, and soft tissue infections—and bacteremia (Holt et al., 2005; Janda, 2014). *Shewanella putrefaciens* is also known as an important spoilage bacterium of seafood (Jia et al., 2018; Liu et al., 2018; Jia et al., 2019). Previous studies have reported *Shewanella* spp. infections in several aquatic animals such as European sea bass (*Dicentrarchus labrax*), loach (*Misgurnus anguillicaudatus*), common carp (*Cyprinus carpio*), rainbow trout (*Oncorhynchus mykiss*), goldfish (*Carassius auratus auratus*), European eel (*Anguilla anguilla*), American eel (*Anguilla rostrata*), and tilapia (*Oreochromis niloticus*) (Kozinska and Pekala, 2004; Korun et al., 2009; Lu and Levin, 2010; Qin et al., 2014; Turgay et al., 2014; Pękala et al., 2015; Wang et al., 2020). The infected tilapia showed skin hemorrhage, fin rot and shallow necrotizing ulcers on the skin with congestion and enlargement of the liver, spleen and kidneys (Manal, 2017). However, the role of *S. putrefaciens* in causing diseases in aquaculture is poorly understood.

Largemouth bass (*Micropterus salmoides*) are native to North America. In the past few years, this species has become an economically important freshwater aquaculture fish in China (Bai et al., 2008; Li et al., 2017). China produced 619, 519 t of largemouth bass in 2020, increased almost 30% of its total production than that in 2019. The occurrence of disease outbreaks in Chinese aquacultures has become more frequent in recent years, causing considerable damage to the aquaculture of largemouth bass (Ma et al., 2013). In March 2021, largemouth bass being cultured in Henan Province experienced persistent mortalities at a water temperature of approximately 10°C. The clinical symptoms of the affected individuals were indicative of a bacterial disease. In this study, we isolated a strain of *S*. *putrefaciens* (termed XX2021) from diseased largemouth bass and identified it as the main pathogen. We also evaluated the sensitivity of XX2021 to 12 antibiotics and examined the histopathological changes in the intestines, head kidney, liver, and spleen in diseased fish. To the best of our knowledge, this is the first study to report *S*. *putrefaciens* as a pathogen of largemouth bass. Our results provide a reference for the prevention and treatment of the diseases caused by *S*. *putrefaciens* infection in largemouth bass under low temperatures.

## Materials and methods

### Fish

Thirty diseased fish (100 ± 12 g) cultured in three ponds (13,333 m^3^/pond; 40,000–45,000 fish/pond) were randomly selected for this study. The fish were transported in ice boxes to the laboratory for diagnosis and anesthetized with MS-222, following which the pathogen was isolated. Healthy fish (n = 360, 15 ± 2 g) were purchased from a local bass farm. Largemouth bass were acclimatized in aerated tanks at 25 ± 2°C for 14 d. The water temperature was decreased by 2°C per day until it reached 10°C or 18°C. The lethal dose 50 (LD_50_) of the pathogen (XX2021) was measured at different water temperatures. For tissue sampling, fish were anaesthetized with MS-222 (40 mg/L, Sigma-Aldrich).

### Isolation and identification of the pathogen

Bacteria were isolated from spleen and muscle tissue of diseased largemouth bass. The isolates were streaked on LB agar medium, followed by incubation at 28°C for 36 h, and the dominant isolated bacteria were re-streaked on the medium three times.

Total genomic DNA was extracted from isolates using bacterial DNA extraction kits (Sangon Biotech, China). The universal primers (27F: 5’-AGAGTTTGATCCTGGCTCAG-3’ and 1492R: 5’TACGGCTACCTTGTTACGACTT-3’) were used for PCR amplification of the bacterial 16S rRNA gene (Weisburg et al., 1991).

Selected 16S rRNA sequences of *Shewanella* spp. were aligned using CLUSTAL multiple alignment in MEGA 5.0. The neighbor-joining method was used to construct a phylogenetic tree after bootstrapping (10,000 replicates). The JTT matrix-based method was used for tree construction. All positions in the alignment that contained gaps or eliminated data after pairwise sequence comparisons (pairwise deletion) were removed, as described previously (Jiang et al., 2016).

### Phenotypical characterization

The physiological and biochemical identification of XX2021 was performed as described previously (Yuan et al., 2021). The Biolog GenIII MicroPlate technique (protocol B, Biolog) was used for this experiment, and final results were recorded at 24 and 48 h of incubation. The physiological and biochemical characteristics of XX2021 could be identified by the phenotypic fingerprint shown on the Biolog GenIII MicroPlate system. The morphology of the bacteria was examined with Gram staining and observation under a scanning electron microscope with the method discribed by Aid et al. (2020). The hemolytic activity of the bacteria was determined using the blood agar plate method. Briefly, the bacteria were inoculated on the medium containing 5% sheep blood. After 36 h of incubation at 28°C, hemolytic isolates were identified based on the presence of α- or β-hemolysis around the colonies.

### Antimicrobial sensitivity testing

Antimicrobial sensitivity was tested as previously described (Pei et al., 2021). In brief, *S*. *putrefaciens* strain XX2021 was spread on Muller–Hilton agar medium, and different kinds of antimicrobial discs were placed on the streaked cultures. Discs containing streptomycin (10 μg), sulfamethoxazole (25 μg), florfenicol (30 μg), doxycycline (30 μg), tetracycline (30 μg), kanamycin (30 μg), gentamicin (10 μg), nalidixic acid (30 μg), norfloxacin (10 μg), cefotaxime (30 μg), ampicillin (10 μg), and penicillin (10 μg) were used. After 24 h of incubation at 28°C, the diameters of inhibition zones around the discs were measured. The bacteria were classified as susceptible (S), intermediate susceptible (I), or resistant (R), based on the guidelines of the National Committee for Clinical Laboratory Standards.

### Infection test and detection of virulence genes

To determine whether the disease was caused by XX2021, we performed an infection test in healthy largemouth bass as described previously (Pei et al., 2021). Bacterial concentrations were determined by plating 10-fold serial dilutions of the isolates onto LB plates. To examine the LD_50_ of XX2021 at different water temperatures, largemouth bass were randomly divided into three groups (120 fish/group) and acclimatized to different temperatures. Each group was further divided into six subgroups, including five infection subgroups and one control subgroup (20 fish/subgroup). Infection groups were injected intraperitoneally with XX2021 at a concentration of 10^2^–10^6^ CFU/g fish weight in 10-fold increments. The control groups were injected with an equal dose of sterile phosphate-buffered saline. The experiment was performed twice. The mortality of fish was monitored for 7 d, and LD_50_ was determined using probit regression analysis in SPSS 20.0 (SPSS Inc., IBM Corp., USA). The bacteria were re-isolated and re-identified from infected largemouth bass. Twelve potential virulence genes in *Shewanella* spp. were detected from the genomic DNA of XX2021 with specific primers (**Table 1**) (Saticioglu et al., 2021).

**Table 1:** Primers for virulence gene testing.

Genes	Primer sequences (5’–3’)	Excepted size/bp
nqrF-F	CGCCTTAGCGAGTCAAAGTA	446
nqrF-R	CATTCACGGTAGAAACGATGT	
lon-F	CGAGATGTGGTGGTCTATCC	326
lon-R	TTGTCTTCTAAAGGCTCTGATT	
atpA-F	TCGGTGCCGTAGTAATGGGTC	192
atpA-R	CAGGTGCAATCACTTCAACAGGAG	
guaA-F	AACTGATTGCCCGTCGTATC	346
guaA-R	GGTTTCCCTTCGCTATTGAC	
luxS-F	TGCTCCTGCGGTACGTGTTG	254
luxS-R	CTTGATGCTCGGTTGGCTCT	
crp-F	AAGGCGACTTTATTGGTGAG	205
crp-R	AAATTGCCGACTTTCTGACT	
dksA-F	ACGCTAAGCAACTGGGTCAC	252
dksA-R	AGGAATCACAGAAGCCGAAA	
exu-F	TAGTGCCCGCCATCGGTGAA	66
exu-R	TACCGTTCCATTGGCTAAAA	
hem-F	CCACAGTGTTAGCCATTCAG	254
hem-R	TGATAGTCACGCATAGCCAC	
fur-F	CTGCTCGATATTGGTGAAGA	165
fur-R	GCCACAGGATAGGCAAACTA	
ompR-F	GTCAACAAGGTAGCCTCATTCC	258
ompR-R	CATCACCGTGGTACATTTCG	
fla-F	ACTCAGCCTGTAATGATGTAA	172
fla-R	CGTAACGCTAACGATGGTAT	

### Histological examination

Histological examination was performed as described previously (Pei et al., 2021). Tissues collected from the spleen, liver, head kidney, and intestines of control and XX2021-injected fish were fixed with 4% paraformaldehyde for 12 h, submerged in 70% alcohol, dehydrated in tertiary butyl alcohol (10%–100%), and embedded in paraffin. Thereafter, paraffin-embedded tissues were cut into 5-μm-thick sections using a microtome (Thermo HM340E, American) and then stained with hematoxylin and eosin. The histological slides were examined with a light microscope (Olympus BX51, Japan).

## Results

### Clinical signs and symptoms

**Figure 1:**
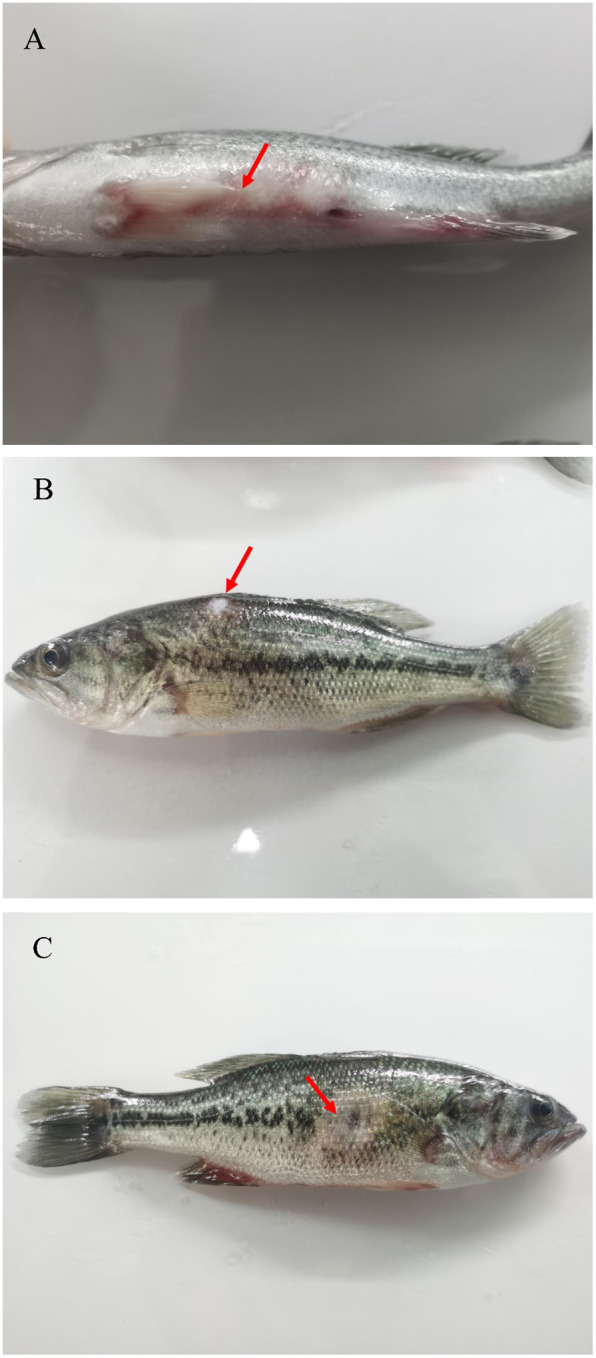
Clinical symptoms of XX2021-infected largemouth bass. **(A)** Hemorrhage in the abdomen (red arrow); **(B)** Ulceration on the back (red arrow); **(C)** Ulceration on the abdomen (red arrow).

In diseased largemouth bass obtained from the local farm, the clinical symptoms included hemorrhage in the abdomen (**Figure 1A**) and ulceration on the back and abdomen (**Figures 1B, C**). The diseased fish also showed appetite loss and sluggish behavior (slow swimming and weak reactions to stimuli). Healthy largemouth bass infected with the isolated bacteria showed symptoms similar to the infected fish in the natural.

### Morphological and molecular characteristics of the bacterial pathogen

**Figure 2:**
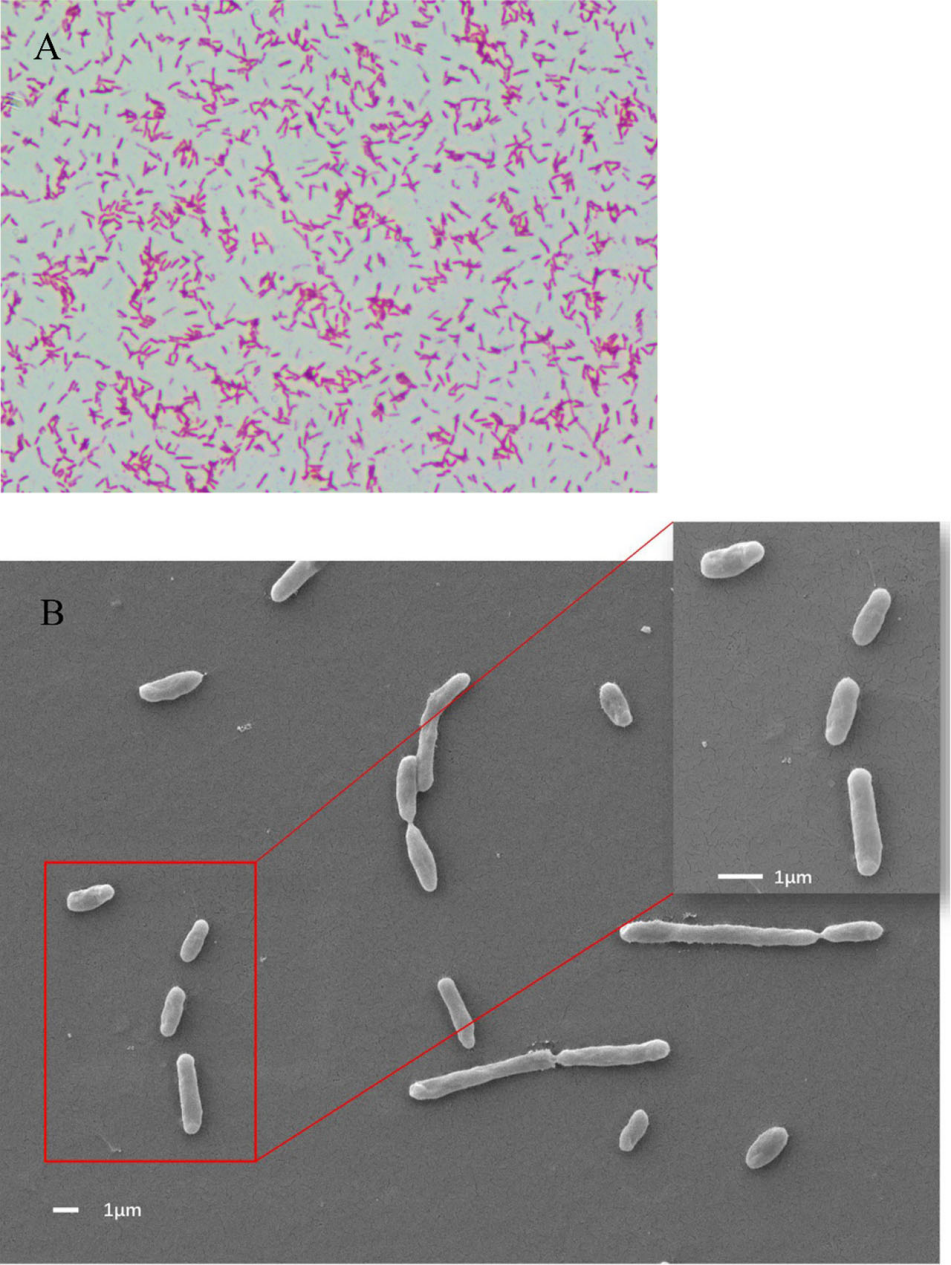
Morphology of XX2021 observed under an optical microscope at 100× magnification and a scanning electron microscope. **(A)** Bacteria (XX2021) were found to be gram-negative under optical microscopy; **(B)** Most isolated bacteria were rod-shaped and lacked a flagellum. The diameter of XX2021 was approximate 0.8 μm.

**Figure 3:**
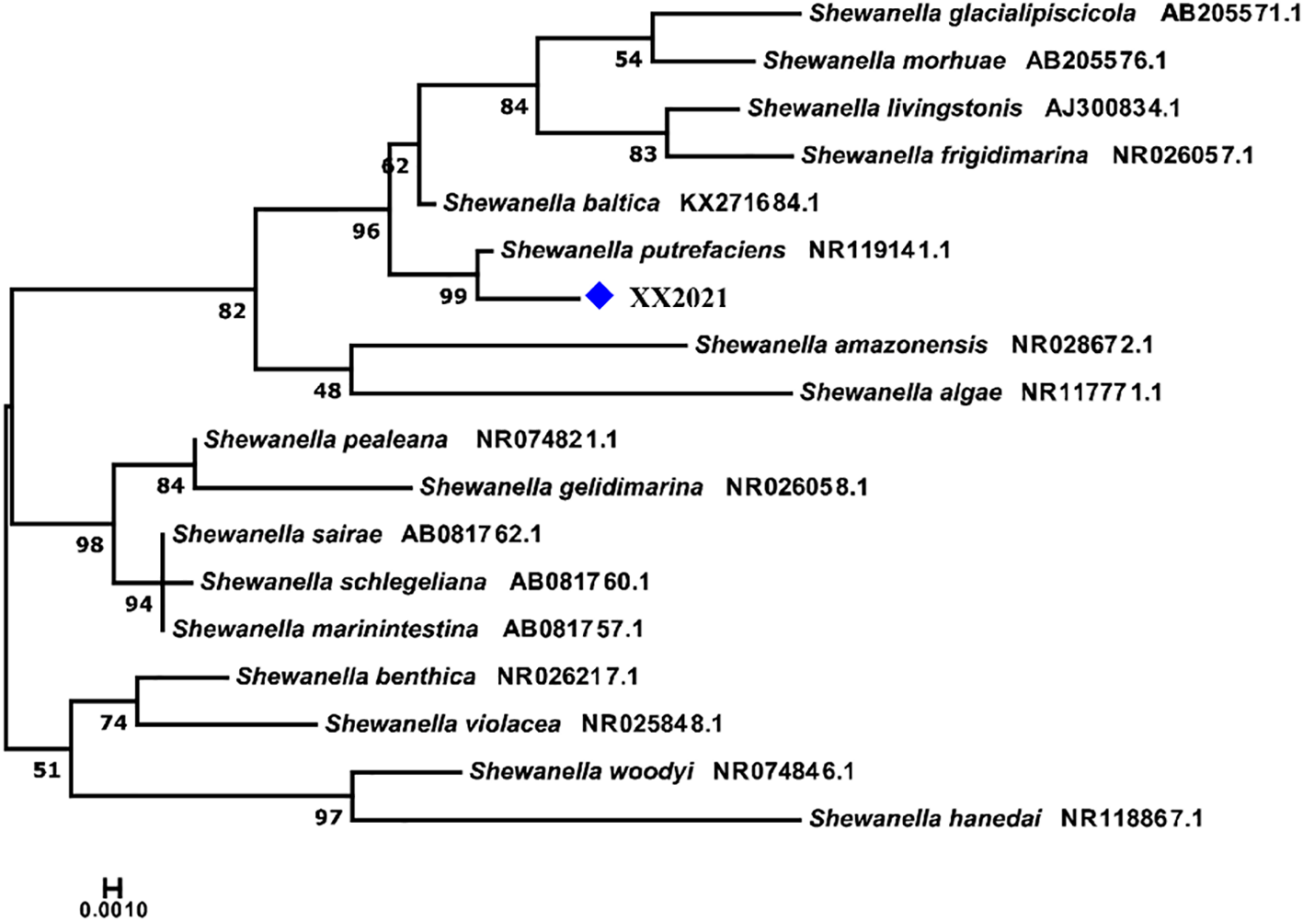
Neighbor-joining (NJ) phylogenetic tree constructed based on the 16S rRNA nucleotide sequences of *Shewanella* species. The percentage of bootstrap values is shown next to the branches based on 10,000 bootstrap replications. The XX2021 sequence is indicated by the blue square.

Upon Gram staining, the isolated bacteria (XX2021) were found to be gram-negative under optical microscopy (**Figure 2A**). When observed under a scanning electron microscope, most isolated bacteria were rod-shaped and lacked flagella. The diameter of the isolated bacterium was approximately 0.8 μm (**Figure 2B**). The colonies on LB plates were buff, circular, convex, and light red in color. The BLAST analysis revealed that the 16S rRNA gene of XX2021 shared the highest similarity (99.7%) with that of the *S*. *putrefaciens* strain (accession number: NR119141.1). A phylogenetic tree was constructed based on the 16S rRNA amino acid sequences of *Shewanella* spp., and XX2021 clustered together with *S*. *putrefaciens* (accession number: NR119141.1) (**Figure 3**).

The physiological and biochemical test results indicated that the bacterial isolate showed positive biochemical reactions for fructose and could grow on a substrate containing 1% (but not 8%) NaCl. It showed negative reactions for raffinose, glucose, sorbitol, rhamnose, and inosine. The substrate utilization patterns and biochemical profiles of XX2021 and *S. putrefaciens* are listed in **Table 2**. Based on these results, we identified the isolated bacteria (XX2021) as *S. putrefaciens*.

**Table 2:** Physiological and biochemical characteristics of strain XX2021.

Characteristics	XX2021	*S*. putrefaciens*
Raffinose	–	–
Rhamnose	–	–
Glucose	–	–
Sorbitol	–	–
Melibiose	V	–
Arginine	V	–
Fructose	+	+
Inosine	–	–
D-Maltose	–	NT
D-Cellobiose	+	NT
Acetic Acid	+	NT
Mannose	NT	+
Aesculin	NT	–
0% NaCl	NT	–
1% NaCl	+	NT
8% NaCl	–	NT
9% NaCl	NT	+
pH6	–	NT

“+”, postivie; “–” negative; V, variable; NT, not tested. * From Qin et al. (2014).

### Evaluation of virulence at different temperatures and analysis of virulence genes

**Figure 4:**
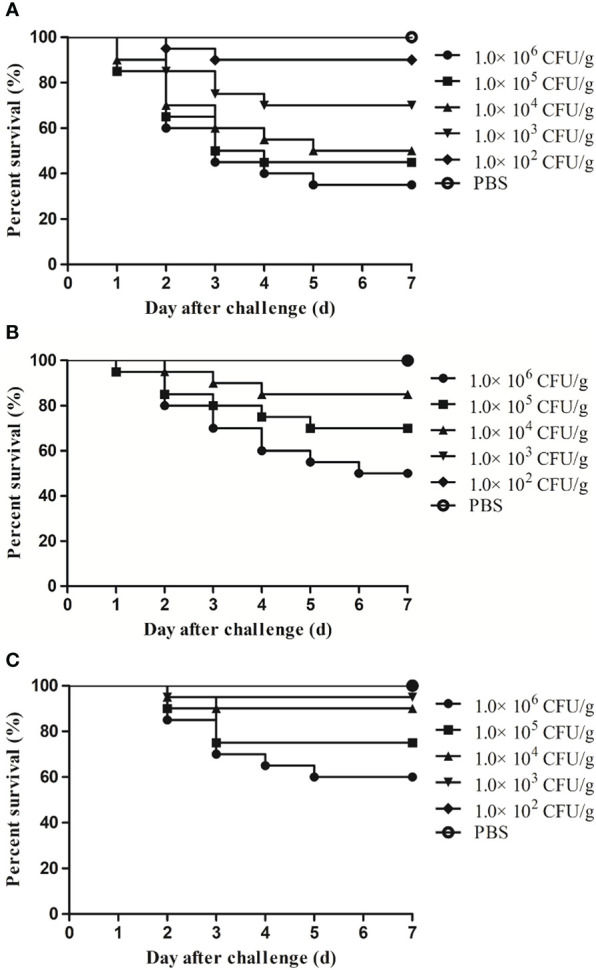
Survival rates of largemouth bass challenged by various doses of XX2021 at different water temperatures during the first 7 days post infection. **(A)** 10°C; **(B)** 18°C; **(C)** 25°C.

**Figure 5:**
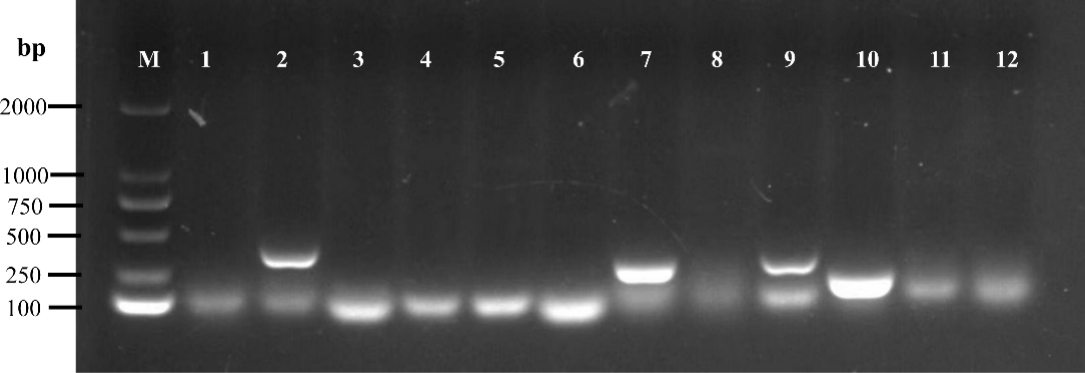
Agarose gel electrophoresis of the PCR products of virulence genes. M: DL2000 marker. Lane 1: *nqr*F, 2: *lon*R, 3: *atp*A, 4: *gua*A, 5: *lux*, 6: *crp*, 7: *dks*A, 8: *exu*, 9: *hem*, 10: *fur*, 11: *omp*R, 12: *fla*.

To examine the effect of temperature on the virulence of XX2021, we performed an infection test in healthy largemouth bass. Following injection with the pathogen, the fish mostly died on days 2–6. The highest cumulative mortality rate was 65% when fish were injected with bacteria (concentration, 10^6^ CFU/g fish weight) at 10°C (**Figure 4**). The LD_50_ of XX2021 was 4.21×10^4^, 7.26×10^5^, and 2.47×10^6^ CFU/g fish weight at 10, 18, and 25°C, respectively. We also identified 12 potential virulence genes using the PCR method; the virulence genes *lon*R*, dks*A*, hem*, and *fur* were screened out (**Figure 5**). The isolates did not exhibit α- or β-hemolysis on sheep blood agar (**Figure S**).

### Determination of antibiotic resistance

We examined antimicrobial resistance and susceptibility patterns of XX2021 by measuring the diameter of the zone of inhibition around each antibiotic disc. The isolate was susceptible (S) to cefotaxime, sulfamethoxazole, florfenicol, doxycycline, tetracycline, kanamycin, and gentamicin; showed intermediate susceptibility (I) to ampicillin, norfloxacin, and streptomycin; and was resistant (R) to nalidixic acid and penicillin (**Table 3**).

**Table 3:** Antibiotic susceptibility profiles of isolated *S. putrefaciens.*.

Antimicrobial	Disc content	Mean Inhibition Zone	Sensitivity
	(μg)	Diameter (mm)	
Cefotaxime	30	40.0	S
Sulfamethoxazole	25	35.0	S
Florfenicol	30	35.0	S
Doxycycline	30	30.0	S
Tetracycline	30	23.0	S
Kanamycin	30	22.0	S
Gentamicin	10	20.0	S
Ampicillin	10	16.0	I
Norfloxacin	10	16.0	I
Streptomycin	10	14.0	I
Nalidixic acid	30	13.0	R
Penicillin	10	0.0	R

S, Susceptible; I, Intermediate susceptible; R, Resistant.

### Histopathology

**Figure 6:**
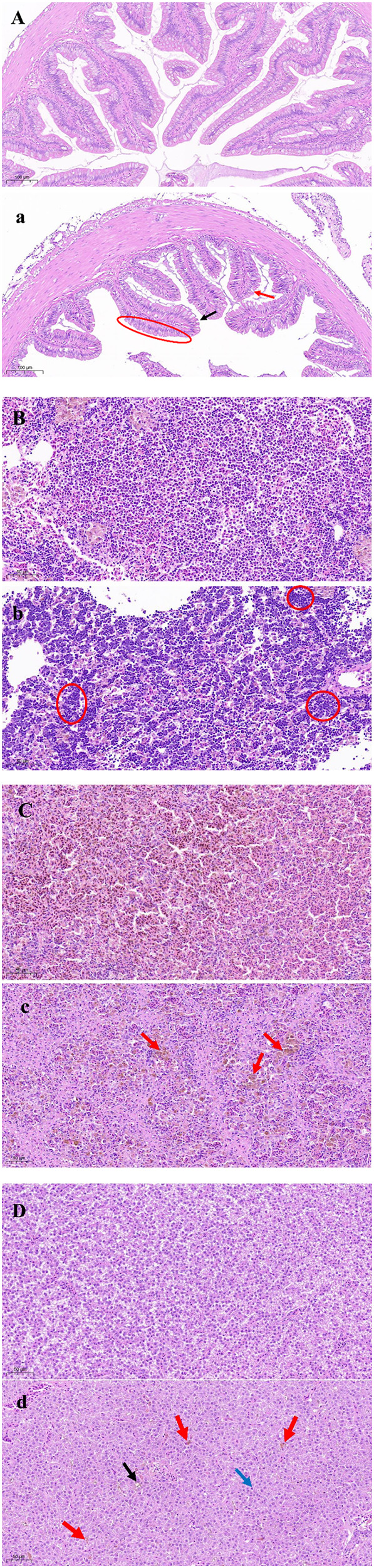
Histological changes in largemouth bass challenged by the XX2021 strain. **(A-D)** are the intestines, head kidney, spleen, and liver of a healthy fish, respectively; **(a)** Goblet cell absence (black arrow), epithelium vacuolization (red arrow), and cell degeneration and vagueness of the cuticula (the area circled in red) in the intestine. **(b)** Head kidney infiltrated by numerous inflammatory cells (the area circled in red). **(c)** Abundant hemosiderin granules (red arrows) in the spleen. **(d)** Serious vacuolization of cells (black arrow), clearly pyknotic nuclei (blue arrow), and an increased number of Kupffer cells (red arrows) in the liver.

Histopathological examination showed that the intestines, head kidney, spleen, and liver exhibited well-organized structures in the control group (**Figures 6A–D**). However, the XX2021-infected group had fewer goblet cells, and exhibited cell degeneration and epithelium vacuolization with vagueness of the cuticula in the intestine (**Figure 6a**). Numerous inflammatory cells had infiltrated the head kidney (**Figure 6b**), and abundant hemosiderin granules (yellow-brown pigment) were observed in the spleen of infected fish (**Figure 6c**). The tissues of infected fish also showed serious vacuolization of cells, clearly pyknotic nuclei, and an increased number of Kupffer cells in the liver (**Figure 6d**).

## Discussion

In this study, we isolated and identified a bacterium (XX2021) from diseased largemouth bass. Physiological and biochemical analyses demonstrated that XX2021 had phenotypic properties consistent with those of *S. putrefaciens*. This is the first recording of a *S. putrefaciens* strain as a pathogen of largemouth bass. The XX2021 isolate was gram-negative, and most cells were rod-shaped and lacked a flagellum (**Figures 1A, B**). *Shewanella putrefaciens* strains isolated from seawater samples in Denmark have been characterized as non-fermentative bacilli with a single polar flagellum (Holt et al., 2005). A highly pathogenic bacterium (AP629) belonging to *Shewanella* spp. was isolated from a sea cucumber (*Apostichopus japonicus*), and these cells were also characterized as short bacilli with a single polar flagellum (Li et al., 2010). Notably, *S. putrefaciens* strains with a flagellum have been detected at water temperatures above 13°C (Gram et al., 1999). In humans, *Shewanella* infections are most common during especially warm summers or in countries with a warm climate (Holt et al., 2005). In this study, XX2021 was isolated from largemouth bass acclimatized to a water temperature of approximately 10°C. Our results are consistent with those of Manal (2017), who isolated a strain of *S. putrefaciens* from *O. niloticus* during the winter season. Moreover, XX2021 was isolated from fresh water and could grow in 1% (but not 8%) NaCl (**Table 2**). In contrast, *S. putrefaciens* strains with a flagellum have been isolated from seawater, which has a higher NaCl concentration than freshwater (Holt et al., 2005). These results indicate that *S. putrefaciens* strains isolated from freshwater or seawater may have different phenotypes due to adaptation to their living environment.

*Shewanella putrefaciens* is a type of zoonotic pathogen. In humans, the most common clinical symptom of *S. putrefaciens* infection occur on the skin or in soft tissue (Ryan et al., 2018; Patel et al., 2020; Benaissa et al., 2021). In this study, infected largemouth bass exhibited several clinical symptoms, including loss of appetite, slow swimming, ulceration on the back and abdomen, and hemorrhage in the abdomen (**Figure 1**). Similar symptoms have also been reported in tilapia, carp, goldfish, and trout infected by *S. putrefaciens* (Kozinska and Pekala, 2004; Turgay et al., 2014; Manal, 2017).

The antimicrobial susceptibility test showed that XX2021 was susceptible to sulfamethoxazole, tetracycline, gentamicin, and florfenicol. Similar results havebeen reported by Altun et al. (2014) and Manal (2017). A strain of *S. putrefaciens* isolated from diseased sea bass was found to be resistant to sulfonamides and tetracycline (Korun et al., 2009). Moreover, although a strain of *S. putrefaciens* isolated from tilapia showed susceptibility to norfloxacin and streptomycin (Manal, 2017), XX2021 showed intermediate susceptibility to these two antibacterial agents. XX2021 also showed complete resistance to both nalidixic acid and penicillin, Therefore, these anti-infection agents should be withdrawn from the therapeutic plan of fish infected by *S. putrefaciens*.

*Shewanella putrefaciens* is a pathogenic bacterium that has been isolated from both marine and freshwater fish (Korun et al., 2009; Qin et al., 2014). Infected fish exhibit emaciation, a distended anus, ulceration on the back and abdomen, and hemorrhage in the abdomen (Pękala et al., 2015). However, one strain of *S. putrefaciens* (SpPdp11) isolated from the skin of healthy gilthead seabream (*Sparus aurata*) has been used as a probiotic. Dietary administration of live SpPdp11 has been shown to significantly increase the expression levels of IgM and protease, the activity of cellular peroxidase, and respiratory burst activity (Varela et al., 2010; Tapia-Paniagua et al., 2014; Cerezuela et al., 2016; Cámara-Ruiz et al., 2020).

Globally, *S. putrefaciens* is known as an important spoilage microorganism of fish during cold storage (Yan and Xie, 2020). Here, we found that the LD_50_ of XX2021 was related to the water temperature, such that the virulence of the isolate was higher at 10°C than at 25°C. This suggests that temperature is a key factor affecting the virulence of *S. putrefaciens*. Similar results have been reported in the channel catfish (*Ictalurus punctatus*), which is farmed in North America. Specifically, *S. putrefaciens* has been shown to infect these fish at water temperatures below 10°C (Mohammed and Peatman, 2018). However, in aquacultures, the optimum temperature for infection by most bacteria is higher than 18°C. Some studies have reported that *S. putrefaciens* can attach to biotic or abiotic surfaces to form biofilms, which contribute to the degradation of seafood quality. The formation of biofilms is promoted at low temperatures, and they protect bacteria from adverse environmental conditions, including the low temperatures (Bagge et al., 2001; Yan and Xie, 2020). These findings suggest that *S. putrefaciens* can infect fish at low water temperatures.

In this study, we detected 12 potential virulence genes in *Shewanella* spp. Of these, four were found in the genome of XX2021. Thereinto, *dksA* encods the RNA polymerase binding transcription factor, and *fur* regulates the intracellular iron transport, storage and utilization in Gram-negative bacteria. These virulence genes provide the basis for follow-up studies on vaccine development and pathogenesis research. In addition, the tissues of XX2021-injected largemouth bass showed histopathological changes in the intestine, head kidney, spleen, and liver (**Figure 6**). Tissue degeneration was likely caused by bacterial toxins. Kupffer cells are involved in the pathogenesis of liver injury caused by bacterial infection (Su, 2002). We found that the number of Kupffer cells in the liver was significantly higher after the injection of XX2021. Similar results have also been reported by Manal (2017). Additionally, the infected fish showed hemosiderin accumulation in the spleen. This may have been because the destruction of blood cells leads to the release of hemoglobin, thus increasing the level of hemosiderin (Qamar et al., 2020). Similar histological findings have been reported in rabbitfish (*Siganus rivulatus*), tilapia, and sea bass infected with *S. putrefaciens* (Saeed et al., 1990; Korun et al., 2009; Manal, 2017; Sood et al., 2020).

## Conclusions

In this study, we isolated a strain of *S. putrefaciens* (termed XX2021) from diseased largemouth bass. The bacteria infected the fish at low water temperatures and caused high mortality. Our findings provide a basis for the development of effective diagnostic strategies for *S. putrefaciens* infection in aquacultures. In addition, we provide novel insights into the prevention of bacterial diseases at low temperatures. Future studies are needed to better elucidate the pathogenic mechanisms of *S. putrefaciens* at low temperatures.

## Data availability statement

The original contributions presented in the study are included in the article/**Supplementary Material**. Further inquiries can be directed to the corresponding author.

## Ethics statement

The animal study was reviewed and approved by Henan normal university animal ethics committee.

## Author contributions

XJ contributed to conception and design of the study and wrote the first draft of the manuscript. XW and LL collected experimental data. CN performed the statistical analysis. CP and LZ wrote sections of the manuscript. XK guided the experiments. All authors contributed to manuscript revision, read, and approved the submitted version.

## Funding

This study was supported by the National Natural Science Foundation of China (Project No. 32002427), the Henan Province Science and Technology Tackling Plan Project (Project No. 202102110260), and the Key Program of Higher Education of Henan Province (Project No. 21A240001).

## Conflict of interest

The authors declare that the research was conducted in the absence of any commercial or financial relationships that could be construed as a potential conflict of interest.

## Publisher’s note

All claims expressed in this article are solely those of the authors and do not necessarily represent those of their affiliated organizations, or those of the publisher, the editors and the reviewers. Any product that may be evaluated in this article, or claim that may be made by its manufacturer, is not guaranteed or endorsed by the publisher.

## Supplementary material

Supplementary Figure Hemolysis testing of XX2021 on sheep blood agar.
