# Supplementary figures and images for: Identification of Shewanella putrefaciens as a novel pathogen of the largemouth bass (Micropterus salmoides) and histopathological analysis of diseased fish
Source: Front Cell Infect Microbiol. 2022 Oct 17;12:1042977. doi: 10.3389/fcimb.2022.1042977 (PMC9618692; doi:10.3389/fcimb.2022.1042977)

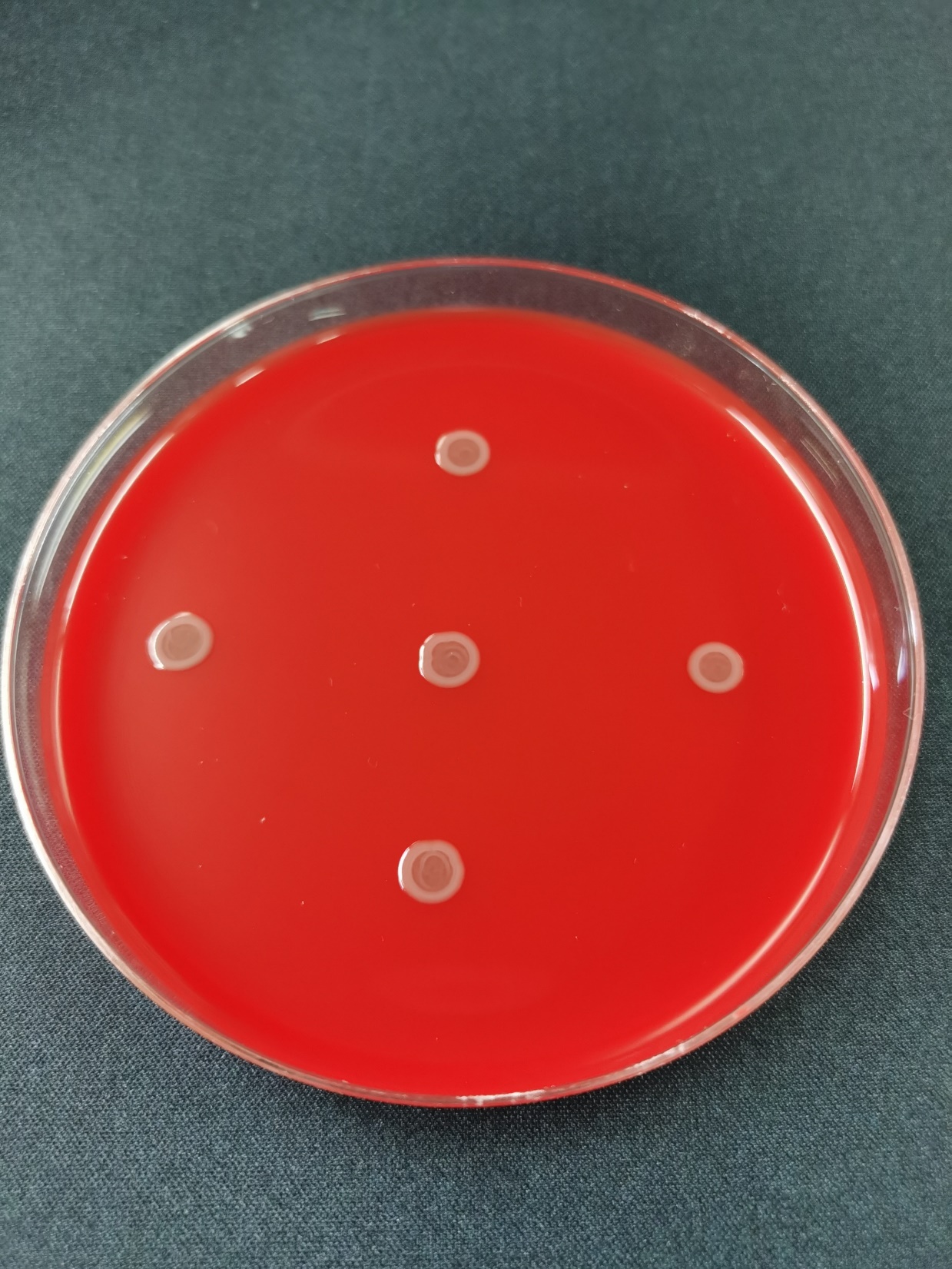
 **Fig. S Hemolysis testing of XX2021 on sheep blood agar.**

Supplement: Supplementary Figure — Hemolysis testing of XX2021 on sheep blood agar. [file DataSheet_1.docx]
